# Left atrium phasic impairments in paroxysmal atrial fibrillation patients assessed by cardiovascular magnetic resonance feature tracking

**DOI:** 10.1038/s41598-022-11233-5

**Published:** 2022-05-09

**Authors:** Mary Luz Mojica-Pisciotti, Roman Panovský, Lucia Masárová, Martin Pešl, Zdeněk Stárek, Tomáš Holeček, Věra Feitová, Lukáš Opatřil, Katarína Doležalová, Vladimír Kincl

**Affiliations:** 1 Cardiovascular Magnetic Resonance Research Group, International Clinical Research Center, St. Anne’s University Hospital, Pekařská 53, 656 91 Brno, Czech Republic; 2 International Clinical Research Center and 1st Department of Internal Medicine/Cardioangiology, St. Anne’s University Hospital, Brno, Czech Republic; 3 Faculty of Medicine, Masaryk University, Brno, Czech Republic; 4 Department of Biology, Faculty of Medicine, Masaryk University, Brno, Czech Republic; 5 International Clinical Research Center and Department of Medical Imaging, St. Anne’s University Hospital, Brno, Czech Republic; 6 Department of Biomedical Engineering, Brno University of Technology, Brno, Czech Republic

**Keywords:** Cardiology, Atrial fibrillation

## Abstract

Atrial fibrillation (AF) is an abnormal and irregular heartbeat caused by uncoordinated electrical impulses in the left atrium (LA), which could induce lasting changes in the heart tissue or could be a consequence of underlying cardiac disease. This study aimed to assess the left atrial phasic function and deformation in paroxysmal AF (PAF) patients—who had not received radiofrequency ablation and had no signs of permanent AF—using the cardiovascular magnetic resonance (CMR) feature-tracking (FT) technique. Fifty subjects (27 PAF patients and 23 controls) were included and examined with CMR. Their LA volume, LA function, LA longitudinal strain (LS) and LA strain rate were assessed in the LA reservoir, conduit, and contractile phases. PAF patients exhibited higher LA volumes than controls, while their LA emptying fraction and LA LS was significantly lower in all three phases. In contrast, the corresponding emptying volumes (total, passive and active) were similar in both groups. The LA volumetric rates from CMR-derived volume curves differed significantly in PAF patients vs controls in the reservoir and contractile phases. In contrast, the equivalent LV volumetric rates were similar. This study suggests that assessing the LA phasic function could offer insight into early LA impairments for PAF patients.

## Introduction

Atrial fibrillation (AF) is the most common cardiac arrhythmia, an abnormal and irregular heartbeat caused by uncoordinated electrical impulses in the atria^1^, which could induce lasting changes in the heart tissue or could be a consequence of underlying cardiac disease^2^. AF may lead to severe complications in heart conditions^1^. This progressive disease worsens with age, manifests a broad range of symptoms, and exhibits a steady increase in its prevalence^2,3^. Paroxysmal AF (PAF), defined as recurrent AF episodes that terminate spontaneously (lasting between 30 s and less than seven days), precedes persistent and permanent AF if left untreated^2^.

The left atrium (LA) function is valuable for understanding the underlying mechanisms associated with AF^2,4,5^. The LA affects left ventricular (LV) filling and ensures optimal cardiac output^6^. Its function divides into three phases: reservoir, when the LA stores pulmonary venous return during LV contraction and isovolumetric relaxation (LV systole); conduit, when the blood is transferred passively into expanding LV (early LV diastole); and contractile, where the LA actively contracts during the final part of LV diastole (just before mitral valve closure)^7^. The LA phase function mainly includes volumetric and volume-derived indexes and deformation imaging parameters. In general, the LA phase function assessment can offer insight into early impairments due to specific pathologies^7–10^, including cardiomyopathy induced by arrhythmias^11^. Some imaging modalities typically used for this purpose are echocardiography-based^4,12^, Doppler-based^4,13^, computed tomography^14^ and cardiovascular magnetic resonance (CMR)^5,7,10,15,16^.

CMR feature tracking (CMR-FT) is an emerging tool that allows a quantitative analysis of regional heart deformation^17,18^. It has a high spatial resolution—for standard steady-state free precession techniques, 1–2 mm in-plane/6–10 mm through-plane^19^—for assessing the LA deformation by tracking the LA wall^5,7–9,16,17,19–22^. A few studies have applied this technique to assess LA function in PAF subjects without any early signs of persistent AF^10,22,23^.

This study aims to compare the LA phase function and deformation in PAF patients—without radiofrequency ablation and no signs of permanent AF—and controls with CMR-FT.

## Methods

### Study population

The Ethics Committee of St Anne's University Hospital Brno approved this prospective study following the Declaration of Helsinki (2000) of the World Medical Association. All the participants were over 18 years, signed informed consent, and had no contraindication for CMR or contrast agents. Fifty subjects (27 PAF patients and 23 controls) were enrolled. The patients were scanned before pulmonary vein ablation and had documented PAF (confirmed by 24-h ECG Holter, with at least one symptomatic episode, EHRA classification II) at the time of the examination. The controls were healthy patients without signs of AF, who had a CMR indication per exclusion of suspected cardiac pathology. Their main indications for CMR examinations were atypical thoracic pain and suspected hypertrophy in those with insufficient echocardiography images. However, they finally had no morphological atrial abnormalities, verified cardiac disease, or CMR findings.

### CMR data acquisition

CMR studies were performed on a 1.5 T scanner (Ingenia, Philips Medical Systems) equipped with 5- and 32-element phased-array receiver coils, allowing parallel acquisition techniques in a supine position with repeated breath-hold. The standard protocol for all the participants included the acquisition of functional imaging with balanced turbo field echo steady-state free precession (SSFP) cine sequences (typical parameters: FOV 300 × 300 mm, acquisition voxel size 1.67 × 1.67 × 8.00 mm, reconstruction matrix 256, slice thickness 8 mm, SENSE factor 1.7, 30 to 50 frames per cardiac cycle). Late gadolinium enhancement (LGE) images were acquired approximately 10 min after a contrast bolus injection [0.2 mmol/kg, gadobutrol (Gadovist, Bayer)]. All participants were in sinus rhythm at the CMR examination to avoid triggering issues. Following the established clinical analysis, an expert radiologist assessed the LV function with the IntelliSpace Portal (ISP) workspace (version 11, Philips Healthcare).

### LA function

LA volumes (LAV) were measured at LV end-systole (maximum LAV, i.e., LAVmax), before the atrial contraction (pre-atrial contraction volume, i.e., LAVpac), and at late LV diastole (minimum LAV, i.e., LAVmin); and indexed to the body surface area (BSA). We assessed the LA function according to^4,13^:

#### Reservoir

$$ {\text{LA}}\,{\text{total}}\,{\text{emptying}}\,{\text{volume }}\left( {{\text{ml}}} \right) \, = {\text{ LAVmax }}{-}{\text{ LAVmin,}} $$

$$ {\text{LA}}\,{\text{total}}\,{\text{emptying}}\,{\text{fraction}}\left( \% \right) \, = { 1}00 \, \times \, \left( {{\text{LAVmax }}{-}{\text{ LAVmin}}} \right)/{\text{LAVmax,}} $$

$$ {\text{LA}}\,{\text{expansion}}\,{\text{index }}\left( \% \right) \, = { 1}00 \, \times \, \left( {{\text{LAVmax }}{-}{\text{ LAVmin}}} \right)/{\text{LAVmin}}{.} $$

#### Conduit

$$ {\text{LA}}\,{\text{passive}}\,{\text{emptying}}\,{\text{volume }}\left( {{\text{ml}}} \right) \, = {\text{ LAVmax }}{-}{\text{ LAVpac,}} $$

$$ {\text{LA}}\,{\text{passive}}\,{\text{emptying}}\,{\text{fraction }}\left( \% \right) \, = { 1}00 \, \times \, \left( {{\text{LAVmax }}{-}{\text{ LAVpac}}} \right)/{\text{LAVmax,}} $$

$$ {\text{LA}}\,{\text{conduit}}\,{\text{volume }}\left( {{\text{ml}}} \right) \, = {\text{ LV}}\,{\text{stroke}}\,{\text{volume }}{-} \, \left( {{\text{Vmax }}{-}{\text{ Vmin}}} \right). $$

#### Contractile

$$ {\text{LA}}\,{\text{active}}\,{\text{emptying}}\,{\text{volume }}\left( {{\text{ml}}} \right) \, = {\text{ LAVpac }}{-}{\text{ LAVmin,}} $$

$$ {\text{LA}}\,{\text{active}}\,{\text{emptying}}\,{\text{fraction }}\left( \% \right) \, = { 1}00 \, \times \, \left( {{\text{LAVpac }}{-}{\text{ LAVmin}}} \right) \, /{\text{ LAVpac}}{.} $$

### CMR-based strain

Two experienced readers assessed the LA longitudinal strain (LS), i.e., a parameter that reflects the deformation of the LA, using long-axis (two-chamber, four-chamber) cine images. They analyzed the images in Image Arena software (2D CPA MR, TomTec Imaging Systems GmbH, v4.6.4.40). Each reader manually traced the LA wall contour in the end-diastole (ED) and end-systole (ES) frames, excluding the pulmonary veins and atrial appendage. The software automatically propagated these contours throughout the cycle and applied the tracking algorithm. The trace accuracy was visually validated and, if necessary, corrected (up to three adjustments per case). In the event of significant suboptimal tracking, the readers repeated the analysis to minimize variability. Each calculation was done three times per view and averaged for improving reproducibility.

**Figure 1:**
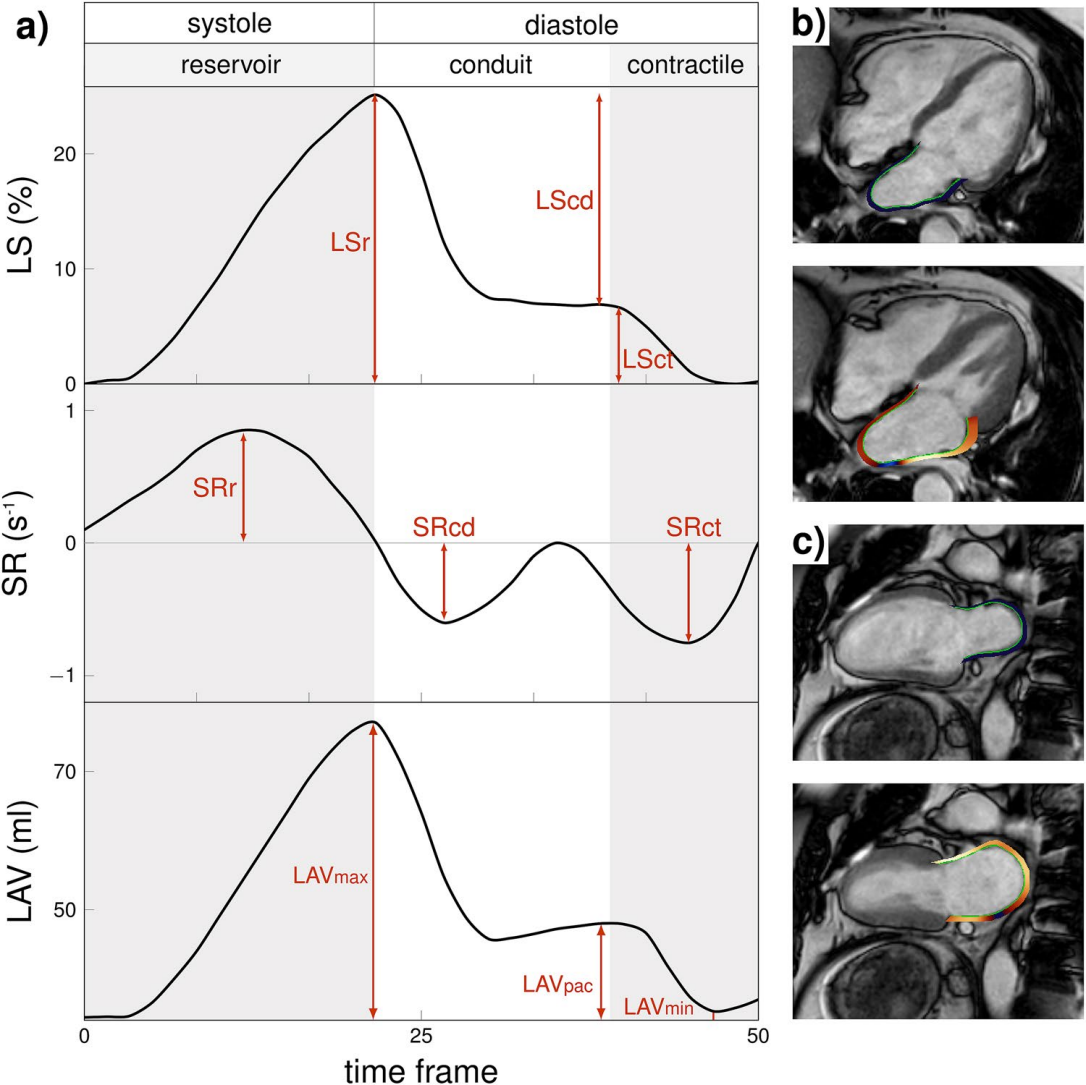
Representation of left atrial function parameters. (**a**) Illustrative representation of the left atrium (LA) strain (LS), strain rate (SR) and volume (V) with corresponding values according to the LA phase, measured with CMR-FT in a control subject. Example of one LA strain analysis performed in (**b**) four-chamber and (**c**) two-chamber views. Upper panel: Left ventricle (LV) end-diastole frame, lower panel: LV end-systole frame. *cd* conduit phase, *ct* contractile phase, *CMR* cardiovascular magnetic resonance, *FT* feature tracking, *LA* left atrium, *LS* longitudinal strain, *LV* left ventricle, *pac* pre-atrial contraction, *SR* strain rate, *r* reservoir phase.

The LA LS was calculated by manually adjusting the specific phases for each view as recommended^24^. LS and strain rate (SR), i.e., the rate at which the LA deforms, are reported for all LA phases: (1) reservoir (LSr, SRr), (2) conduit (LScd, SRcd), and (3) contractile (LSct = LSr − LScd, SRct). From the LA SR curve, the positive peak corresponded to SRr, the early negative to SRcd, and the late negative to SRct (see Fig. 1).

Similarly, for LV strain assessment [global longitudinal strain (GLS), global circumferential strain (GCS) and global radial strain (GRS)], the same readers used long-axis (two-chamber, three-chamber, four-chamber) and short-axis (basal, mid-ventricular, apical) cine images. They followed a similar process as previously reported^25^.

### LA volumetric rates

**Figure 2:**
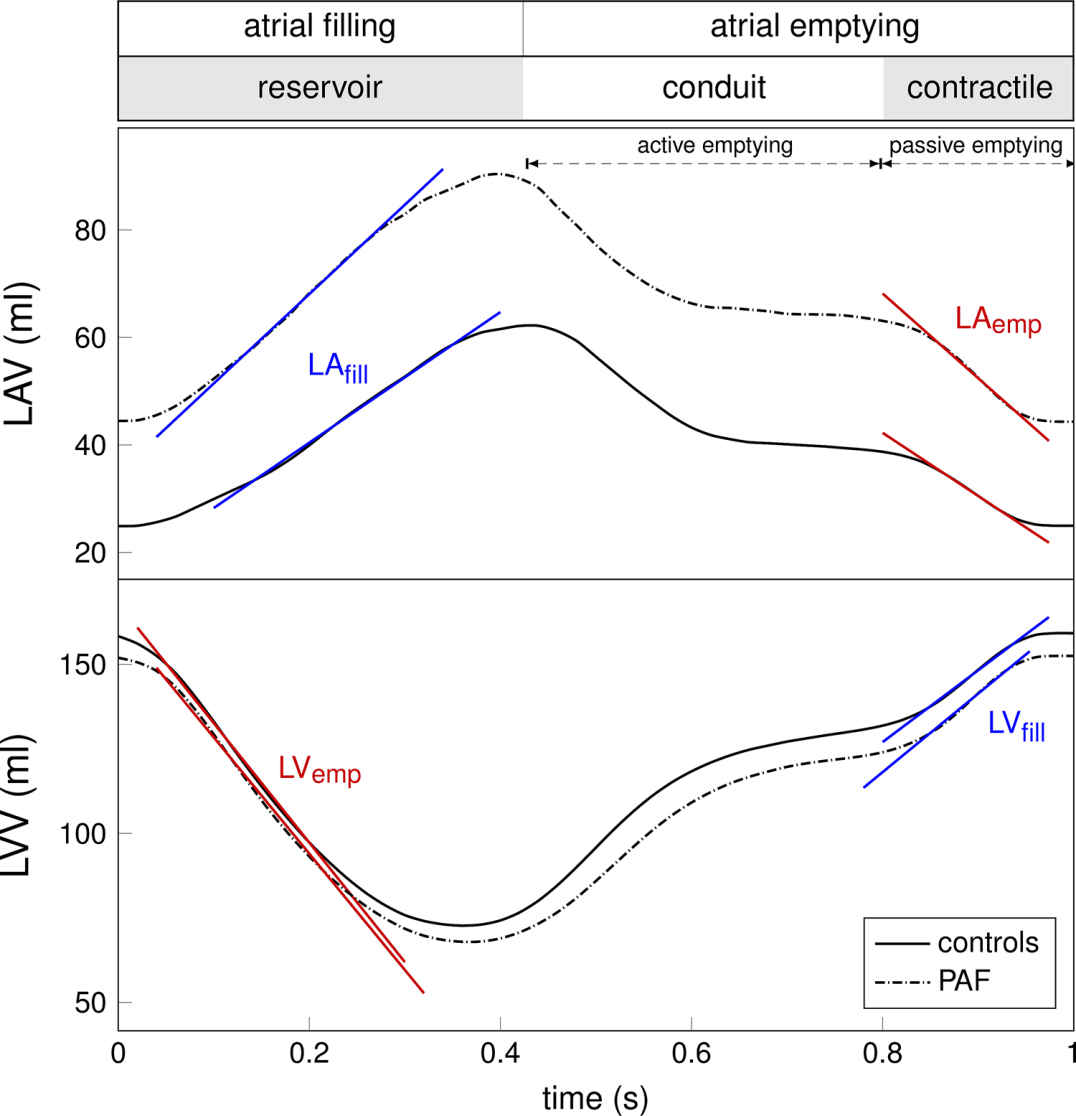
Volume curves derived from CMR-FT. Schematics of the volume vs time curve slope calculation for the left atrium (LA) (upper panel, LAV) and left ventricle (LV) (lower panel, LVV) indicated phases. The curves shown are the average of the controls' (solid) and the PAF patients (dash-dotted) measurements. *CMR* cardiovascular magnetic resonance, *FT* feature tracking, *LA* left atrium, *LA*_*emp*_ LA volume emptying, *LA*_*fill*_ LA volume filling, *LAV* LA volume, *LV* left ventricle, *LV*_*emp*_ LV volume emptying, *LV*_*fill*_ LV volume filling, *LVV* LV volume.

After performing the CMR-based strain analysis of long-axis cine images, global volume vs time curves for the LA and LV were automatically obtained. From them, slopes in the reservoir (LV_emp_ and LA_fill_) and contractile (LV_fill_ and LA_emp_) phases were calculated with a custom-designed tool in Python 3.8.8 (see Fig. 2). The slope values represent the rate of change in the respective volumes, i.e., how fast the volume (ml) changes in a time interval (s).

### Statistical analysis

Descriptive statistics are reported as the mean (SD) or median (IQR) for normally and non-normally distributed continuous variables, respectively, and as numbers (percentages) for categorical ones. The normality of the data was checked by the Shapiro–Wilk test and visual inspection of the histograms. Proportions of categorical variables were analyzed using the Chi-square test of independence. The student's t-test and Wilcoxon rank-sum test were used to compare normally and non-normally distributed variables, respectively. A *P*-value < 0.05 was considered statistically significant. Correlations were calculated using Pearson. The intraobserver and interobserver agreement was assessed with the intraclass correlation coefficient (ICC). The ICC (type C, two-way mixed-effects model) was determined from ten randomly selected cases analyzed by two readers (MLMP, TH), one of whom repeated them one month apart. The repeatability was classified as poor (< 0.5), fair (0.50–0.75), good (0.75–0.90) and excellent (0.90–1)^26^. All statistical analyses were performed with R-4.0.3 and RStudio IDE (v1.3.1093, RStudio, PBC).

## Results

### Group characteristics

Fifty CMR examinations from 27 PAF patients and 23 controls, who met the inclusion criteria, were successfully analyzed. The general characteristics, including the LV function and strain, were similar in both groups (see Table 1). All PAF patients were receiving anticoagulation according to the individual CHADS2-VASc score (mean score for males and females was 1.3 ± 1.3 and 2.4 ± 1.0, *P* = 0.028). The average duration of the history of PAF was 17.0 ± 14.1 months. Twenty-six PAF subjects had antiarrhythmic therapy, alone [beta-blocker alone (n = 6), propafenone alone (n = 1), amiodarone alone (n = 3)] or in combination [beta-blocker with amiodarone (n = 5) or with propafenone (n = 11)], and one patient had not because of bradycardia adverse effects. Sixteen PAF patients had hypertension, and two (non-hypertense) had positive LGE in the LV (intramural inferolateral basal and basal-mid segments, non-ischemic in both cases). There were no significant differences between the LV function or the LV global strain between both groups.

**Table 1:** General characteristics and comparison of left ventricular function and global strain between PAF patients and controls.

	Controls (N = 23)	PAF patients (N = 27)	P-value
Gender (males, %)	7 (30%)	16 (59%)	0.080
Age (years)	59.8 (7.7)	63.0 (6.1)	0.112
BMI (kg/m^2^)	26.1 (3.5)	27.1 (3.3)	0.305
BSA (m^2^)	1.9 (0.2)	2.0 (0.2)	0.090
HR (bpm)	63.1 (9.5)	60.9 (9.1)	0.410
Hypertension (n, %)	8 (35%)	16 (59%)	0.084
Systolic BP (mmHg)	136.8 (19.8)	140.2 (17.6)	0.597
Diastolic BP (mmHg)	84.0 (10.9)	82.0 (9.5)	0.577
Mean BP (mmHg)	110.4 (12.8)	111.1 (11.0)	0.862
Diabetes mellitus, (n, %)	1 (4.3%)	1 (3.7%)	> 0.999
Hypertension (n, %)	9 (39%)	16 (59%)	0.156
Current smoker (n, %)	2 (15%)	5 (19%)	> 0.999
LV EF (%)	70.6 (6.4)	70.1 (8.2)	0.810
LV EDVI (ml/m^2^)	54.6 (7.4)	58.6 (10.9)	0.137
LV ESVI (ml/m^2^)	16.1 (4.8)	17.9 (7.0)	0.294
LV SVI (ml/m^2^)	38.5 (5.4)	40.7 (6.6)	0.212
LVMI (g/m^2^)	41.2 (11.0)	47.3 (12.2)	0.069
GLS (%)	− 20.3 (3.0)	− 20.7 (3.7)	0.680
GCS (%)	− 24.5 (4.3)	− 25.2 (5.0)	0.600
GRS (%)	64.7 (14.7)	60.9 (13.3)	0.340

Variables are expressed as numbers (percentages), mean (standard deviation) or median (interquartile range) for categorical, normally distributed, and non-normally distributed continuous variables, respectively. *BMI* body mass index, *BP* blood pressure, *BSA* body surface area, *HR* heart rate, *LV* left ventricle, *EF* ejection fraction, EDV, end-diastole volume, *ESV* end-systole volume, *GLS* global longitudinal strain, *GCS* global circumferential strain, *GRS* global radial strain, *I* indexed, *LVM* left ventricular mass, *n* number of subjects, *PAF* paroxysmal atrial fibrillation, *SV* stroke volume.

### LA function

The total, passive and active emptying volumes were similar for both groups, but the corresponding emptying fractions were impaired in PAF patients (see Table 2). Additionally, PAF subjects showed significantly higher LA volumes in all LA phases. The LA expansion index (reservoir) was also significantly lower for PAF.

**Table 2:** Comparison of left atrial function between PAF patients and controls.

	Controls (N = 23)	PAF patients (N = 27)	P-value
LAVImax (ml/m^2^)	34.4 (10.2)	45.2 (12.5)	**0.001**
LAVIpac (ml/m^2^)	23.1 (8.4)	33.1 (10.4)	**< 0.001**
LAVImin (ml/m^2^)	13.3 (8.6–16.4)	20.0 (16.7–24.3)	**< 0.001**
**Reservoir phase**			
LA total emptying volume (ml/m^2^)	20.8 (5.4)	23.4 (5.4)	0.093
LA total emptying fraction (%)	62.4 (9.0)	53.4 (9.0)	< **0.001**
LA expansion index (%)	168.7 (142.7–208.4)	119.9 (85.3–151.2)	**0.001**
**Conduit phase**			
LA passive emptying volume (ml/m^2^)	6.2 (2.2)	6.2 (2.0)	0.967
LA passive emptying fraction (%)	34.2 (7.7)	27.2 (7.0)	**0.002**
LA conduit volume (ml/m^2^)	17.7 (6.0)	17.3 (4.2)	0.755
**Contractile phase**			
LA active emptying volume (ml/m^2^)	5.2 (2.4)	5.6 (1.5)	0.433
LA active emptying fraction (%)	42.8 (10.0)	36.0 (8.7)	**0.014**

Significant values are in bold.

Variables are expressed as mean (standard deviation) or median (interquartile range) for normally and non-normally distributed continuous variables. *I* indexed, *LA* left atrium, *LAV* LA volume, *max* maximum, *pac* pre-atrial contraction, *PAF* paroxysmal atrial fibrillation, *min* minimum.

**Figure 3:**
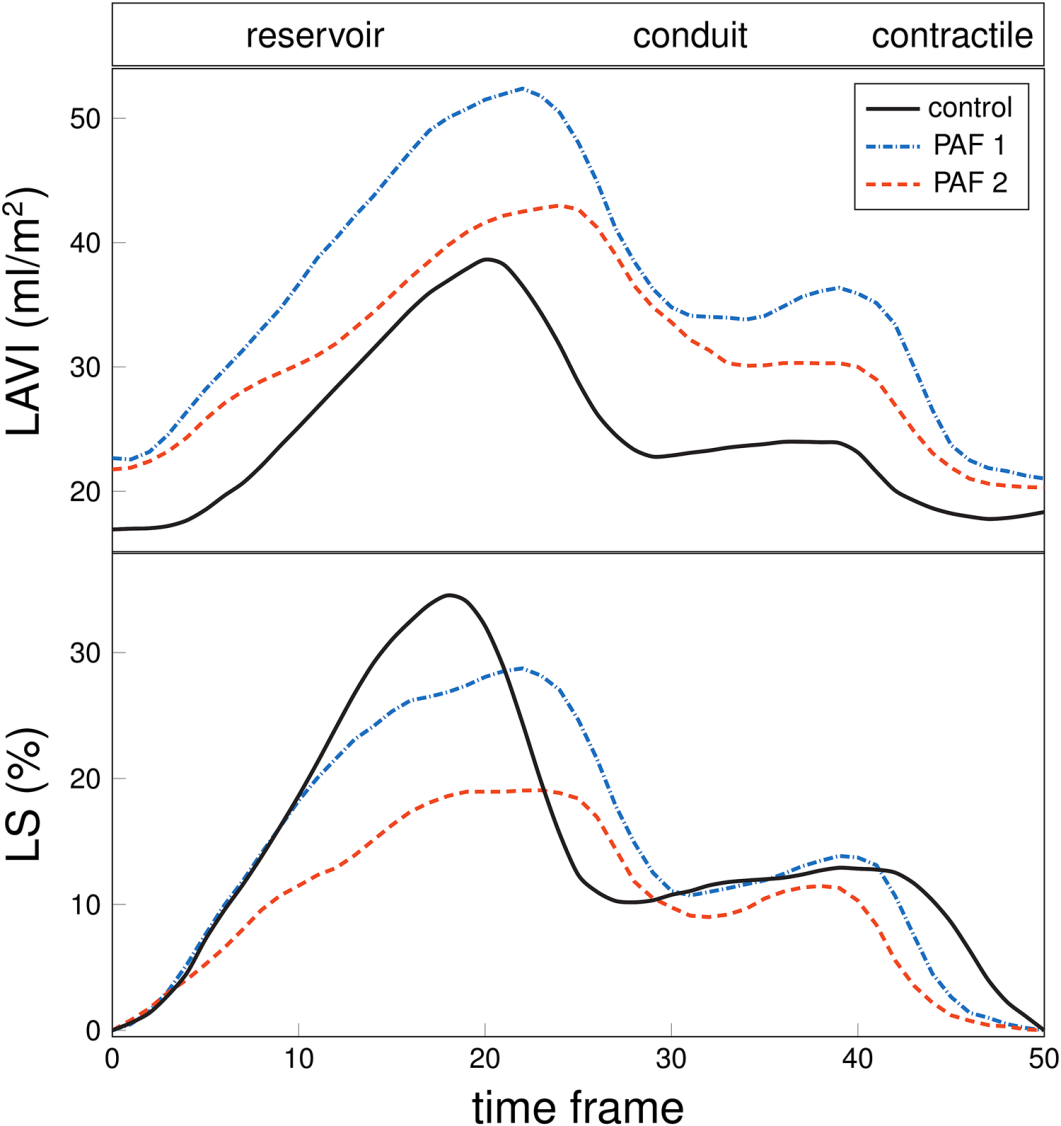
Example of indexed left atrium volume (LAVI) and longitudinal strain (LS) measurements. Example of the LAVI (upper panel) and LA LS (lower panel) measured with CMR-FT in a control subject and two random PAF patients. *CMR* cardiovascular magnetic resonance, *FT* feature tracking, *LA* left atrium, *LS* longitudinal strain.

The LA volumes correlated with the LA strain: r =  − 0.66, *P* < 0.001 (LAVImax and LSr), r =  − 0.49, *P* = 0.018 (LAVIpac and LScd), and r =  − 0.60, *P* = 0.002 (LAVImin and LSct); and with the LA strain rate: r =  − 0.49, *P* = 0.018 (LAVImax and SRr), r = 0.60, *P* = 0.002 (LAVIpac and SRcd), and r = 0.62, *P* = 0.002 (LAVImin and SRct). Figure 3 (upper panel) shows the measurement of LA indexed volumes of three subjects (one control, two PAF patients).

### LA volumetric rates

The LA volumetric rates (LA_fill_ and LA_emp_) were significantly different for both groups in the reservoir and contractile phases (see Table 3). The slope values represent the rate of change of the respective volume change: PAF patients have a significatively higher LA filling volume rate (LA_fill_) and lower LA emptying volume rate (LA_emp_) than controls. A negative slope means the volume of blood decreases with time.

**Table 3:** Comparison of LV and LA absolute value of slopes derived from the corresponding volume vs time curves during the LA reservoir and contractile phases for PAF patients and controls.

	Controls (N = 23)	PAF patients (N = 27)	P-value
**LA reservoir phase**			
LA_fill_ (ml/s) (+)	101.4 (22.8)	129.1 (32.6)	**< 0.001**
LV_emp_ (ml/s) (−)	280.3 (62.1)	311.3 (79.2)	0.128
**LA contractile phase**			
LA_emp_ (ml/s) (−)	116.2 (40.4)	154.1 (54.0)	**0.007**
LV_fill_ (ml/s) (+)	190.0 (62.8)	199.4 (75.6)	0.631

Significant values are in bold.

The sign indicates the blood volume increases (filling) or decreases (emptying) with time. Variables are expressed as mean (standard deviation). *LA* left atrium, *LA*_*emp*_ LA volume emptying, *LA*_*fill*_ LA volume filling, *LV* left ventricle, *LV*_*emp*_ LV volume emptying, *LV*_*fill*_ LV volume filling, *PAF* paroxysmal atrial fibrillation.

There were significant negative correlations for the volumetric rates during the reservoir and contractile phases for PAF patients (reservoir (LV_emp_ and LA_fill_) r =  − 0.54, *P* = 0.003 and contractile (LV_fill_ and LA_emp_) r =  − 0.64, *P* < 0.001) (see Table 3). The equivalent correlations for controls were non-statistically significant.

### LA strain and strain rate

LS was significantly lower for PAF patients than controls in all LA phases (see Table 4). The SR reached statistical significance in the conduit and contractile phases. Figure 3 (bottom panel) shows the LS measurement of three subjects (one control, two PAF patients).

**Table 4:** Comparison of left atrial strain and strain rate values between PAF patients and controls.

	Controls (N = 23)	PAF patients (N = 27)	P-value
LSr (%)	32.7 (28.1 to 39.1)	26.1 (21.5 to 30.5)	**0.002**
LScd (%)	17.6 (13.8 to 23.1)	13.0 (11.9 to 17.2)	**0.040**
LSct (%)	14.9 (12.1 to 19.7)	10.5 (8.8 to 13.3)	**0.003**
SRr (s^−1^)	1.5 (1.3 to 1.9)	1.4 (1.2 to 1.5)	0.070
SRcd (s^−1^)	− 1.4 (− 1.8 to − 1.0)	− 1.1 (− 1.3 to − 0.9)	**0.019**
SRct (s^−1^)	− 1.6 (− 2.3 to − 1.1)	− 1.0 (− 1.4 to − 0.9)	**0.003**

Significant values are in bold.

Variables are expressed as median (interquartile range). *cd* conduit, *ct* contractile, *LS* longitudinal strain, *PAF* paroxysmal atrial fibrillation, *r* reservoir, *SR* strain rate.

### Reproducibility

LAV and LS measurements had an excellent intra- and interobserver agreement (ICC > 0.98, see Table 5). The coefficients were good/fair for SR, and the less reproducible parameter was the SRcd.

**Table 5:** Intraobserver and interobserver reproducibility (ICC, two-way mixed-effects model) of LA volumes, strain and strain rate determined from two measurements of ten random subjects.

	Intraobserver		Interobserver	
	ICC	95% CI	ICC	95% CI
LAVmax	0.996	0.985–0.999	0.932	0.766–0.982
LAVpac	0.989	0.960–0.997	0.919	0.724–0.979
LAVmin	0.989	0.961–0.997	0.932	0.766–0.982
LSr	0.991	0.965–0.998	0.996	0.983–0.999
LScd	0.981	0.929–0.995	0.997	0.988–0.999
LSct	0.988	0.956–0.997	0.975	0.908–0.994
SRr	0.915	0.714–0.978	0.843	0.514–0.958
SRcd	0.853	0.539–0.961	0.616	0.054–0.887
SRct	0.943	0.799–0.985	0.885	0.626–0.970

*cd* conduit, *ct* contractile, *CI* confidence interval, *ICC* intraclass correlation coefficient, *LS* longitudinal strain, *SR* strain rate, *r* reservoir.

## Discussion

This study compared the LA function in the reservoir, conduit and contractile phases in PAF patients and controls, assessed by the corresponding emptying volume and fraction, longitudinal strain and strain rate. The PAF patients exhibited impairment in their LA emptying fraction and longitudinal strain in all LA phases. Expectedly, our results indicate that PAF patients have larger LA volumes than controls in all phases, as stated elsewhere^9,23,27^. Several cardiac diseases can manifest through LA size changes: LA enlargement can predict worsening clinical outcomes^8,10,13,20,27–30^, and it is a risk factor for PAF^9,27^.

The LA volumetric rates (LA_fill_ and LA_emp_) suggest a possible compensation mechanism that allows the LA to deform faster or slower enough to receive or release blood volume during the LA phases. In our case, the LA filling (reservoir phase) was faster in PAF patients, indicating that their LA receives more blood in a fixed time interval. Considering the LS is impaired during that phase, the results suggest that the LA cavity could experience pressure or volume overload, allowing the LA enlargement. At the same time, the LA emptying (contractile phase) was slower in PAF patients, which means they release less blood than controls in a given time: the consequence is that the PAF patient's LA holds a higher blood volume during the whole cardiac cycle. However, other explanatory factors should be independently assessed^31^. Similar approaches to studying CMR-based volume rates for diastolic dysfunction have been published and validated^32,33^, advocating for the method as a potential imaging-based marker. The presented LA volumetric approach has not been studied in a PAF cohort, as far as we know.

Of distinct interest for us was studying the LA deformation through the LS, which besides directly measuring the deformation, reflects the electromechanical integrity of the tissue^34^. Our results agree with other CMR-FT based studies, which indicate the LS might decline before other LA functional parameters^5^ and affects all the LA phases^5,23,35^. Although we lack consensus about the influence of each phase in disease development, the weakening affects them in an early stage of AF (when no persistent AF has developed).

We also found a decline in PAF patients' LA conduit and contractile functions (lower passive and active emptying fraction, LS and SR). The LA conduit function could provide insight into the LV relaxation^36^, and the LA contractile function mirrors the LV loading conditions as it directly relates to the LV end-diastole^15^. In addition to the LScd and LSct impairment, SRcd and SRct were higher for PAF patients, reinforcing the idea of a possible LA contractile dysfunction. However, SR results must be interpreted carefully as the CMR temporal resolution is limited.

LA function impairments in patients with PAF have also been reported in other works based on CMR-FT^5,10,23^. However, the cohorts included patients who had not yet developed AF^5^ or patients with hypertrophic cardiomyopathy^23^ or persistent AF^10^. Our work complements these, especially considering that the included PAF patients had no other significant pathologies affecting their heart, did not receive radiofrequency ablation before the CMR exam, and had no persistent or permanent AF signs.

Other authors assessed LA deformation in PAF using echocardiography-derived techniques^4,9,15,31,34,35,37,38^, reporting a reduced LA contractile function^15,31^, extending to LS and SR^35^. LS seems to be lower in patients with PAF and primary arterial hypertension^38^; the frequency and number of PAF episodes might enhance LA reservoir function in these patients^9^, and the LA wall degenerates more in PAF subjects^15^. In many reports, the authors highlight the importance of assessing LA function rather than sole LA size to study and predict possible PAF development^4,9,15,35,37,38^.

CMR-FT and echocardiography-derived techniques show notable differences between the strain and strain rate assessment^39^. However, the LS derived from both methods has good agreement^40^. While CMR-FT has a higher spatial resolution that allows a better LA contouring, echocardiography has a better temporal resolution and is a more non-invasive and simple method^18^. In contrast, unlike echocardiography methods, CMR-FT offers consistent and highly reproducible imaging planes. Both imaging modalities have advantages that might impact a future determination of a gold standard for deformation imaging in PAF.

Finally, the study mainly focused on studying LA phasic function and deformation for a group of PAF diagnosed patients—without radiofrequency ablation and no signs of permanent AF. Our results provide further evidence of the feasibility of using LS in clinical practice to understand PAF evolution, enriching the current perspective.

### Limitations

Our study has some limitations. It was single-centre, and the sample size was relatively small, which impeded a proper predictive risk analysis. We did not stratify the risk of developing diastolic dysfunction. The controls were primarily women, which might impact the results as they have a lower myocardial mass. We presented a simple evaluation of LV and LA volumetric changes using CMR-derived volume vs time curves. However, we lack echocardiographic-based validation in our specific cohort. Although such an approach has been previously validated^32,33,41^, there is no gold standard for these measurements. Besides the complexity of the LA function assessment, the CMR-FT tracking process may be challenging. The LA wall is narrow, which increases the probability of sub-tracking during analysis; it also uses contours but no intra-tissue markers, which exhibits high variability. However, the use of CMR-FT for LS analysis is reasonable and has good reproducibility^8,22^. The SR assessments had lower reproducibility, which could improve by increasing the temporal resolution. Still, it would require longer breath-holds, which the patient cannot consistently maintain in most cases. Although we cannot provide specific ranges of values for pairing patients with PAF development, our results support the potential use of LS in PAF for future advances, such as studying changes in these patients after radiofrequency ablation.

## Conclusion

In conclusion, our results support the potential inclusion of LA phase function assessed by CMR-FT to become a comprehensive marker for PAF patients. We found significant larger LA volumes and decreased LS in PAF patients in all LA phases.

## Data Availability

The datasets generated and analyzed during the current study are available from the corresponding author on reasonable request.

## References

[1] Starek Z, Lehar F, Jez J, Wolf J, Novák M (2015). Hybrid therapy in the management of atrial fibrillation. Curr. Cardiol. Rev..

[2] Iwasaki Y-K, Nishida K, Kato T, Nattel S (2011). Atrial fibrillation pathophysiology. Circulation.

[3] Kornej J, Börschel Christin S, Benjamin Emelia J, Schnabel Renate B (2020). Epidemiology of atrial fibrillation in the 21st century. Circ. Res..

[4] Blume GG (2011). Left atrial function: Physiology, assessment, and clinical implications. Eur. J. Echocardiogr..

[5] Bertelsen L (2020). Left atrial volume and function assessed by cardiac magnetic resonance imaging are markers of subclinical atrial fibrillation as detected by continuous monitoring. EP Europace.

[6] Pagel PS (2003). Mechanical function of the left atrium: New insights based on analysis of pressure-volume relations and doppler echocardiography. Anesthesiology.

[7] Yang Y (2020). Quantification of left atrial function in patients with non-obstructive hypertrophic cardiomyopathy by cardiovascular magnetic resonance feature tracking imaging: A feasibility and reproducibility study. J. Cardiovasc. Magn. Reson..

[8] Funk S (2018). Quantification of the left atrium applying cardiovascular magnetic resonance in clinical routine. Scand. Cardiovasc. J..

[9] Cui Q (2008). Enhanced left atrial reservoir, increased conduit, and weakened booster pump function in hypertensive patients with paroxysmal atrial fibrillation. Hypertens. Res..

[10] Habibi M (2015). Association of left atrial function and left atrial enhancement in patients with atrial fibrillation: Cardiac magnetic resonance study. Circ. Cardiovasc. Imaging.

[11] Huizar JF, Ellenbogen KA, Tan AY, Kaszala K (2019). Arrhythmia-induced cardiomyopathy: JACC state-of-the-art review. J. Am. Coll. Cardiol..

[12] Thomas L (2020). Evaluation of left atrial size and function: Relevance for clinical practice. J. Am. Soc. Echocardiogr..

[13] Hoit BD (2017). Evaluation of left atrial function: Current status. Struct. Heart.

[14] Hansen PB, Sommer A, Nørgaard BL, Kronborg MB, Nielsen JC (2017). Left atrial size and function as assessed by computed tomography in cardiac resynchronization therapy: Association to echocardiographic and clinical outcome. Int. J. Cardiovasc. Imaging.

[15] Hirose T (2012). Left atrial function assessed by speckle tracking echocardiography as a predictor of new-onset non-valvular atrial fibrillation: Results from a prospective study in 580 adults. Eur. Heart J. Cardiovasc. Imaging.

[16] Olsen FJ (2016). Multimodality cardiac imaging for the assessment of left atrial function and the association with atrial arrhythmias. Circ. Cardiovasc. Imaging.

[17] Scatteia A, Baritussio A, Bucciarelli-Ducci C (2017). Strain imaging using cardiac magnetic resonance. Heart Fail. Rev..

[18] Schuster A, Hor KN, Kowallick JT, Beerbaum P, Kutty S (2016). Cardiovascular magnetic resonance myocardial feature tracking. Circ. Cardiovasc. Imaging.

[19] Pedrizzetti G, Claus P, Kilner PJ, Nagel E (2016). Principles of cardiovascular magnetic resonance feature tracking and echocardiographic speckle tracking for informed clinical use. J. Cardiovasc. Magn. Reson..

[20] Hinojar R (2019). Prognostic value of left atrial function by cardiovascular magnetic resonance feature tracking in hypertrophic cardiomyopathy. Int. J. Cardiovasc. Imaging.

[21] Ali RL (2019). Arrhythmogenic propensity of the fibrotic substrate after atrial fibrillation ablation: A longitudinal study using magnetic resonance imaging-based atrial models. Cardiovasc. Res..

[22] Kowallick JT (2014). Quantification of left atrial strain and strain rate using Cardiovascular Magnetic Resonance myocardial feature tracking: A feasibility study. J. Cardiovasc. Magn. Reson..

[23] Sivalokanathan S (2019). Hypertrophic cardiomyopathy patients with paroxysmal atrial fibrillation have a high burden of left atrial fibrosis by cardiac magnetic resonance imaging. JACC Clin. Electrophysiol..

[24] Badano LP (2018). Standardization of left atrial, right ventricular, and right atrial deformation imaging using two-dimensional speckle tracking echocardiography: A consensus document of the EACVI/ASE/Industry Task Force to standardize deformation imaging. Eur. Heart J. Cardiovasc. Imaging.

[25] Panovský R (2021). Quantitative assessment of left ventricular longitudinal function and myocardial deformation in Duchenne muscular dystrophy patients. Orphanet J. Rare Dis..

[26] Koo TK, Li MY (2016). A guideline of selecting and reporting intraclass correlation coefficients for reliability research. J. Chiropr. Med..

[27] Nori D (2009). Cardiac magnetic resonance imaging assessment of regional and global left atrial function before and after catheter ablation for atrial fibrillation. J. Interv. Card. Electrophysiol..

[28] Boyd AC, Thomas L (2014). Left atrial volumes: Two-dimensional, three-dimensional, cardiac magnetic resonance and computed tomography measurements. Curr. Opin. Cardiol..

[29] Khan MA (2019). Association of left atrial volume index and all-cause mortality in patients referred for routine cardiovascular magnetic resonance: A multicenter study. J. Cardiovasc. Magn. Reson..

[30] Gulati A (2013). Clinical utility and prognostic value of left atrial volume assessment by cardiovascular magnetic resonance in non-ischaemic dilated cardiomyopathy. Eur. J. Heart Fail..

[31] Kojima T (2012). Left atrial global and regional function in patients with paroxysmal atrial fibrillation has already been impaired before enlargement of left atrium: Velocity vector imaging echocardiography study. Eur. Heart J. Cardiovasc. Imaging.

[32] Bowman AW, Kovács SJ (2004). Left atrial conduit volume is generated by deviation from the constant-volume state of the left heart: A combined MRI-echocardiographic study. Am. J. Physiol. Heart Circ. Physiol..

[33] Aquaro GD (2019). Diastolic dysfunction evaluated by cardiac magnetic resonance: The value of the combined assessment of atrial and ventricular function. Eur. Radiol..

[34] Park JJ (2020). Left atrial strain as a predictor of new-onset atrial fibrillation in patients with heart failure. JACC Cardiovasc. Imaging.

[35] Kuppahally Suman S (2010). Left atrial strain and strain rate in patients with paroxysmal and persistent atrial fibrillation. Circ. Cardiovasc. Imaging.

[36] Marino PN (2021). Left atrial conduit flow rate at baseline and during exercise: An index of impaired relaxation in HFpEF patients. ESC Heart Fail..

[37] Di Salvo G (2005). Atrial myocardial deformation properties predict maintenance of sinus rhythm after external cardioversion of recent-onset lone atrial fibrillation. Circulation.

[38] Jarasunas J, Aidietis A, Aidietiene S (2018). Left atrial strain—An early marker of left ventricular diastolic dysfunction in patients with hypertension and paroxysmal atrial fibrillation. Cardiovasc. Ultrasound.

[39] Kowallick JT, Lotz J, Hasenfuß G, Schuster A (2015). Left atrial physiology and pathophysiology: Role of deformation imaging. World J. Cardiol..

[40] Kim J (2020). Left atrial strain impairment precedes geometric remodeling as a marker of post-myocardial infarction diastolic dysfunction. JACC Cardiovasc. Imaging.

[41] Duvall W (2013). Validation of real-time 3d echocardiography left ventricular volume-time curves with cardiac MRI and clinical utilization of emptying and filling rates. J. Cardiovasc. Dis. Diagn..

